# Special Issue: Principal Challenges in the Adjuvant Treatment of Glioblastoma

**DOI:** 10.3390/biomedicines11071881

**Published:** 2023-07-03

**Authors:** Marc-Eric Halatsch

**Affiliations:** Department of Neurosurgery, Cantonal Hospital of Winterthur, CH-8400 Winterthur, Switzerland; marc-eric.halatsch@ksw.ch; Tel.: +41-52-266-2974

Despite advances in local treatments, such as supramaximal resection (even in eloquent locations [1]) and guidance of surgery and radiotherapy by metabolic imaging [2], patients with glioblastoma, the most common malignant intrinsic brain tumor in adults, continue to face a dismal prognosis following so-called standard therapy, which has recently evolved to include alternating electric fields [3]. While a plethora of rational experimental strategies for additive therapy is currently under clinical development [4], the failure of new approaches during clinical translation has been the rule rather than the exception [5], with alternating electric fields (a device rather than a drug) being this exception.

Prominent failures include unsuccessful molecular targeting of the epidermal growth factor receptor (EGFR). This approach can achieve promising results in vitro and in some preclinical models [6,7,8,9,10], and EGFR amplification, overexpression, and ligand-independent activation are molecular hallmarks of a substantial fraction of glioblastomas. Nonetheless, this type of therapy has not lived up to its apparent promises [11]. Remarkably, the EGFR has recently been declared a *Lazarus* molecular target in glioblastoma, and draws parallels with the tenacious “undruggability” of oncogenic RAS [12,13]. It would be truly telling if a biomarker-independent combination of multiple, originally non-oncological, repurposed drugs, as well as low-dose metronomic temozolomide [14,15,16], performed better against glioblastoma in the clinical setting than the current molecular targeting of aberrant EGFR.

Until new therapies have been validated, and likely beyond that point, chemotherapy with temozolomide will remain an integral part of postoperative first-line treatment. For this reason, experts are currently taking a closer look at some of the controversies surrounding temozolomide, and this Special Issue is dedicated to their findings. Strobel et al.; Kaina; Stepanenko and Chekhonin; and Westhoff et al. [17,18,19,20] have engaged in a highly instructive scholarly debate on temozolomide’s predominant biological effects under different experimental conditions and how these conditions can be used to optimize modeling of clinical situations in vitro. In this vein, Herbener et al. provide an extensive review of the experimental use of temozolomide in glioblastoma research, concluding that conciliating two ambivalent properties of temozolomide, i.e., immunogenicity and immunosuppression, is among the most challenging issues from both research and clinical perspectives [21].

Last but not least, in a study conducted by Papadopoulos et al., haloperidol, a typical antipsychotic drug that permeates the blood–brain barrier, caused cell cycle arrest and the inhibition of cellular migration in glioblastoma cell lines when combined with radiation and temozolomide in vitro [22], once more highlighting the potential of repurposed drugs to enhance the efficacy of existing treatment regimens.

In summary, this Special Issue brings together a variety of inspiring perspectives on different aspects of this field of inquiry, providing a valuable resource for anyone performing and/or evaluating research on this complex subject. Readers are invited to assess the data and different arguments presented, as these will undoubtedly widen our perspective on the most important issues currently under discussion.
